# Supplementary material for: Coupled small molecules target RNA interference and JAK/STAT signaling to reduce Zika virus infection in Aedes aegypti
Source: PLoS Pathog. 2022 Apr 4;18(4):e1010411. doi: 10.1371/journal.ppat.1010411 (PMC9017935; doi:10.1371/journal.ppat.1010411)
Supplement: S1 Table — (DOCX) [file ppat.1010411.s001.docx]

**S1 Table. Primers for qRT-PCR and siRNA synthesis**

| **Gene** | **Forward qRT-PCR primer** | **Reverse qRT-PCR primer** | **Citation** |
| --- | --- | --- | --- |
| *Ae*Actin | GAACACCCAGTCCTGCTGACA | TGCGTCATCTTCTCACGGTTAG | Diop et al., *Viruses* 2019 |
| *Ae*AGO2 | CAACTTCGGTATCCTTCT | TTCCCGTCTTGTAATCTCC | Bernhardt et al., *PLOS One* 2012 |
| *Ae*Vir-1 | GCCAAAGTCCGGTATTCTTC | TTCACGAGATCGTCAAGGTAA | Diop et al., *Viruses* 2019 |
| *Ae*P400 | GGAACCAGTCCAGCCATGAA | CGATCGCTCCTGCATTTGTG | McFarlane et al., *mSphere* 2020 |
| *Ae*Vago2 | CGACCCGGAATGTGTGAAGA | GCAGCATTGTGGGTAGTCCT | Asad, Parry, and Asgari, *Insect Biochem and Mol Bio* 2018 |
| *Ae*Dicer2 | GTGTAATCGGTCTTTCTG | ACGCCAGTCTTAGCATTG | Bernhardt et al., *PLOS One* 2012 |
| *Ae*Ppo8 | GCTTTGCTATGTCCGCCAAT | CGCATTCGGAGACAATGATG | Almire et al. *PLOS Path* 2021 |
| *Ae*Dome | AAACGGTGGCAAAATGAACT | CATACAGCCGGCTTTCTTCT | Souza-Neto et al., *Proc Natl Acad Sci USA* 2009 |
| *Ae*AGO2 siRNA | \| ACAACAGCAACAATCCCAGA \|  \|  \| \| --- \| --- \| --- \| \|  \|  \| | \| GTGGACGTTGATCTTGTTGG \|  \| \| --- \| --- \| | Terradas, Joubert, and McGraw, *Sci Rep* 2017 |
| *Ae*Vir-1 siRNA | GCCAAAGTCCGGTATTCTTC | TTCACGAGATCGTCAAGGTAA | Terradas, Joubert, and McGraw, *Sci Rep* 2017 |

| **Gene** | **Sense/ Antisense** | **Reverse dsRNA primer** | **Start** | **Target Sequence** | **Citation** |
| --- | --- | --- | --- | --- | --- |
| *Ae*AGO2 | sense | CCUAAAGCAGGGUGUCCAAdTdT | 1836 | CCTAAAGCAGGGTGTCCAA | Terradas, Joubert, and McGraw, *Sci Rep* 2017 |
|  | antisense | UUGGACACCCUGCUUUAGGdTdT | 1836 | TTGGACACCCTGCTTTAGG | Terradas, Joubert, and McGraw, *Sci Rep* 2017 |
| *Ae*Vir-1 | sense | CGGAAGAUACCCAGACCAAdTdT | 404 | CGGAAGATACCCAGACCAA | Terradas, Joubert, and McGraw, *Sci Rep* 2017 |
|  | antisense | UUGGUCUGGGUAUCUUCCGdTdT | 404 | TTGGTCTGGGTATCTTCCG | Terradas, Joubert, and McGraw, *Sci Rep* 2017 |
| *Ae*Scramble | sense | GATTAGACGAATACCACTA |  |  | Clemons et al., *PLOS One* 2011 |
|  | antisense | CTAATCTGCTTATGGTGAT |  |  | Clemons et al., *PLOS One* 2011 |
