# Supplementary figures and images for: Coupled small molecules target RNA interference and JAK/STAT signaling to reduce Zika virus infection in Aedes aegypti
Source: PLoS Pathog. 2022 Apr 4;18(4):e1010411. doi: 10.1371/journal.ppat.1010411 (PMC9017935; doi:10.1371/journal.ppat.1010411)

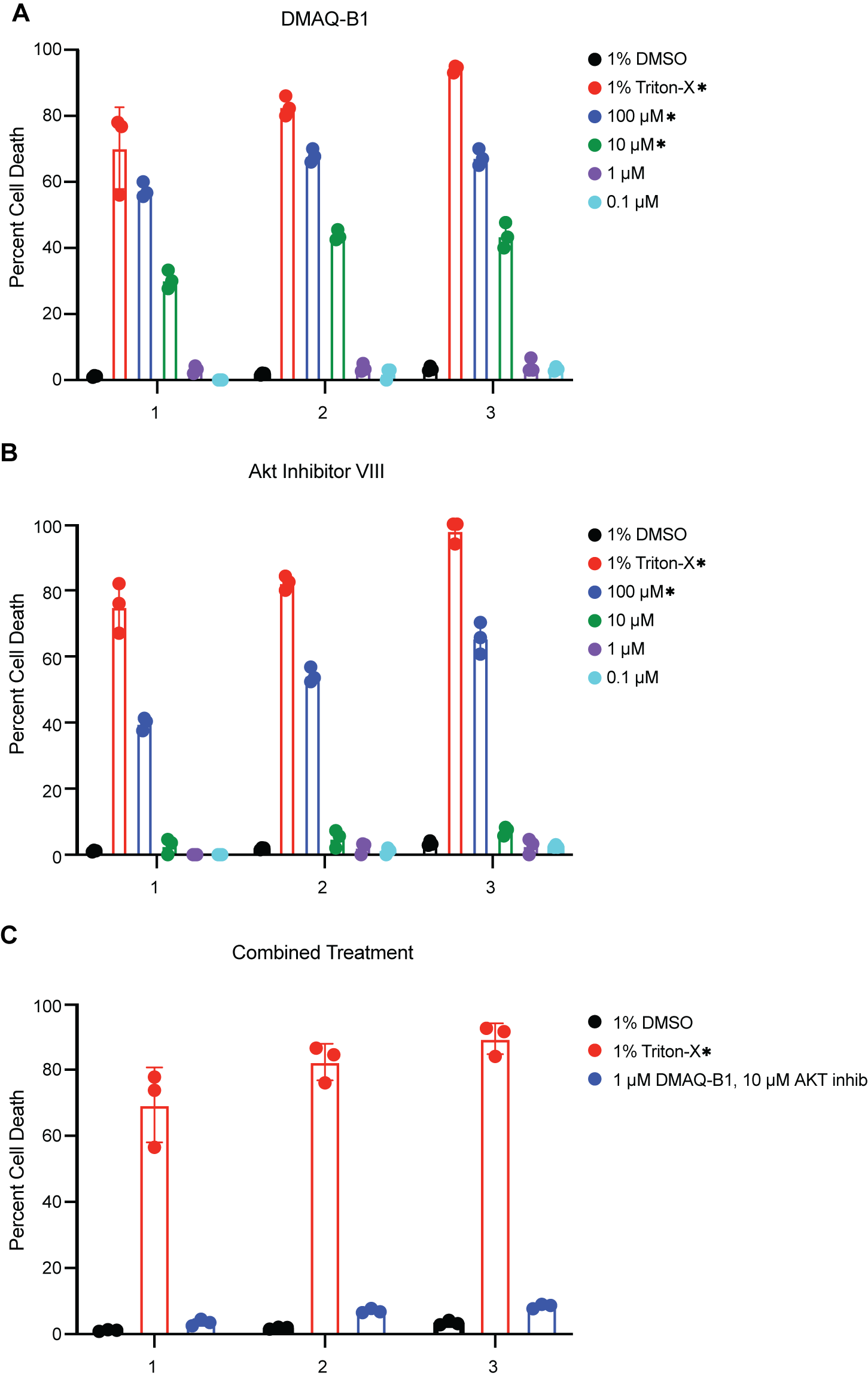

Supplement: S1 Fig — Aag2 cells were treated with various concentrations of (A) DMAQ-B1, (B) AKT inhibitor VIII, (C) combined drugs, or DMSO vehicle control and cell viability was measured by trypan blue exclusion. Cells that received 1% Trixton-X-100 treatment were used as a positive, 100% lethality control. Closed circles represent biological replicates measured in technical triplicate. Horizontal black bars represent the mean. Error bars represent SD. Significance was measured by Two-Way ANOVA with 1% DMSO vehicle control (*p<0.01). Data are representative of triplicate independent experiments. (TIF) [file ppat.1010411.s003.tif]

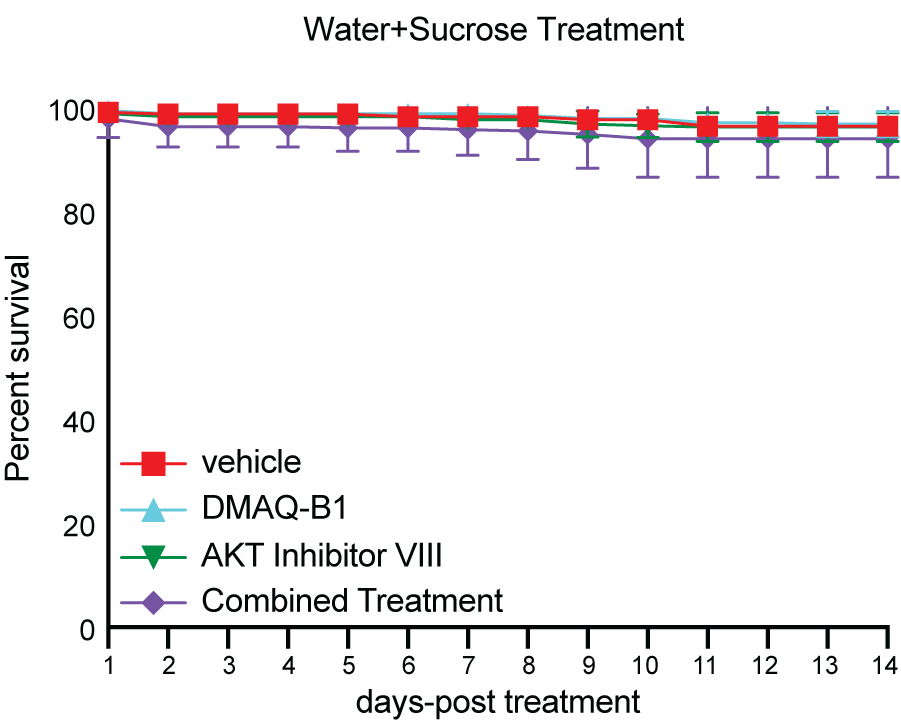

Supplement: S2 Fig — Adult female Ae. aegypti were given sucrose water supplemented with 1% DMSO (vehicle), 10 μM DMAQ-B1, 10 μM AKT inhibitor VIII, or combined drugs ad libitum and toxicity was measured by survival over 14 days. Closed circles represent percent survival of mosquitoes (n = 60–100) measured in triplicate. Horizontal black bars represent the mean. Error bars represent SD. Significance was measured by Two-Way ANOVA with 1% DMSO vehicle control. Data are pooled triplicate independent experiments. (TIF) [file ppat.1010411.s004.tif]

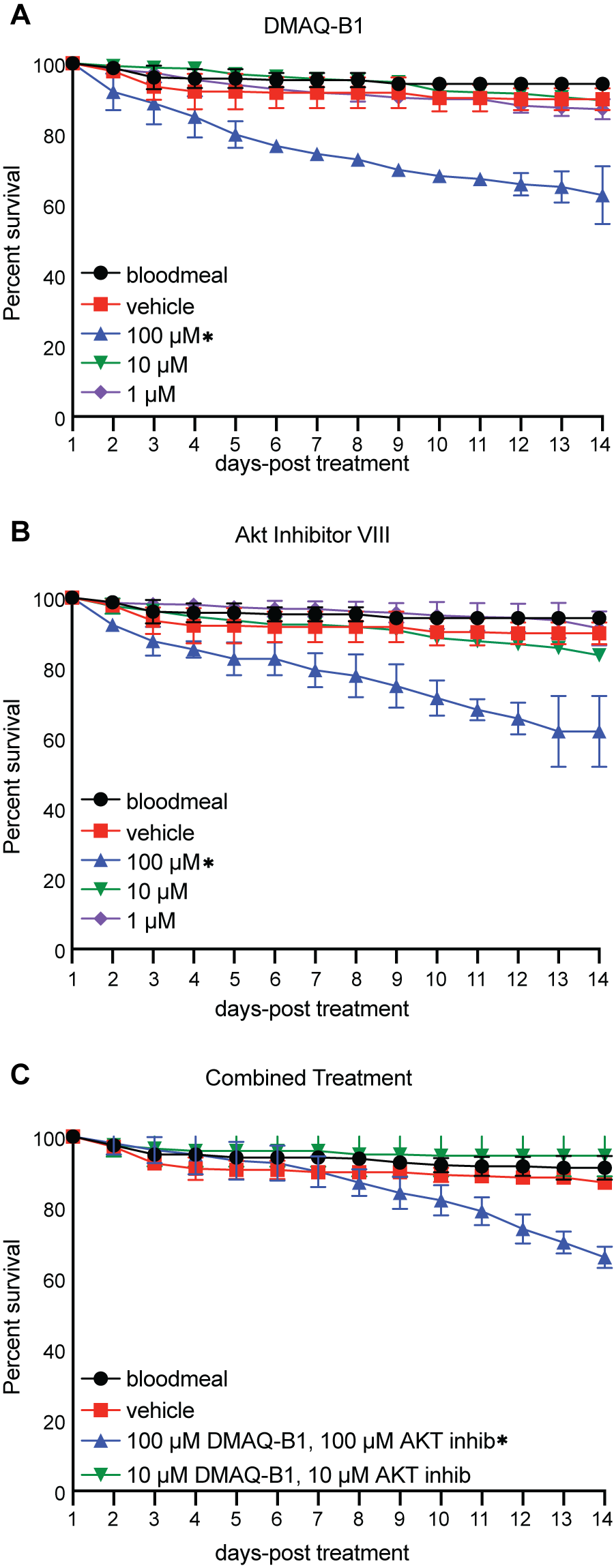

Supplement: S3 Fig — Adult female Ae. aegypti were treated with various concentrations of (A) DMAQ-B1, (B) AKT inhibitor VIII, (C) combined drugs and toxicity was measured by survival over 14 days. Closed circles represent percent survival of mosquitoes (n = 60–100) measured in triplicate. Horizontal black bars represent the mean. Error bars represent SD. Significance was measured by Two-Way ANOVA with 1% DMSO vehicle control (*p<0.05). Data are representative of duplicate independent experiments. (TIF) [file ppat.1010411.s005.tif]

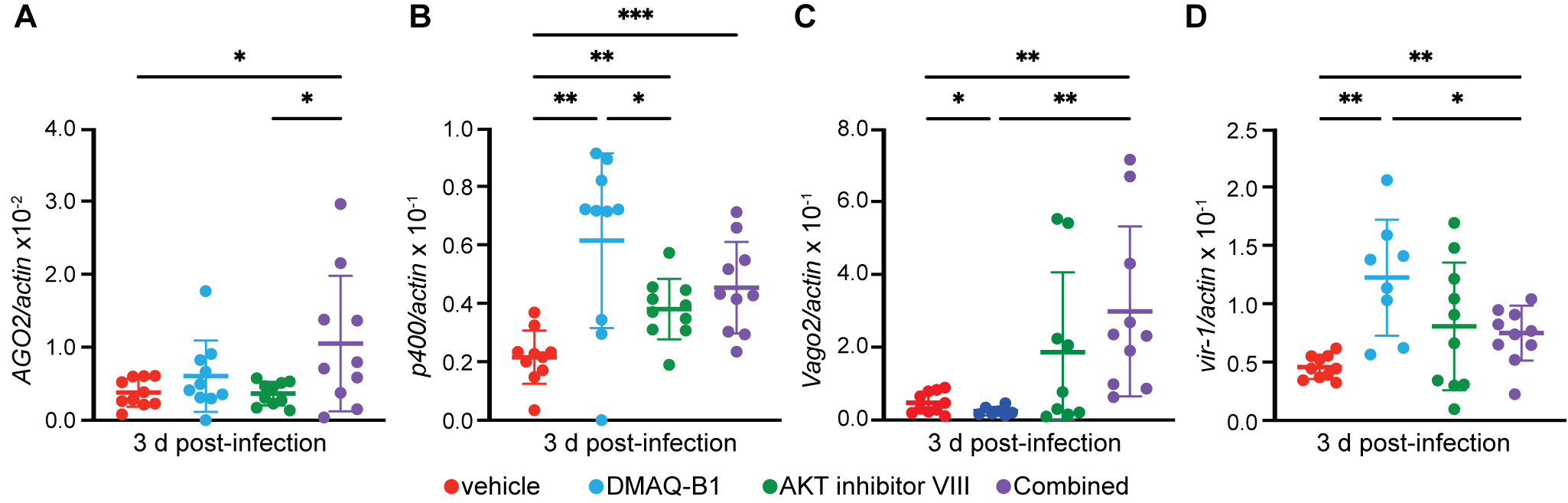

Supplement: S4 Fig — Induction of (A) AGO2, (B) p400, (C) Vago2, and (D) vir-1 in adult female Ae. aegypti was measured by qRT-PCR 3 d p.i. of ZIKV- and drug-containing bloodmeal. (*p<0.05; **p < 0.01; ***p < 0.001). Open circles represent individual biological replicates. Outliers were identified using a ROUT test (Q = 5%) and removed. Horizontal black bars represent the mean. Error bars represent SDs. Data are representative of duplicate independent experiments. (TIF) [file ppat.1010411.s006.tif]

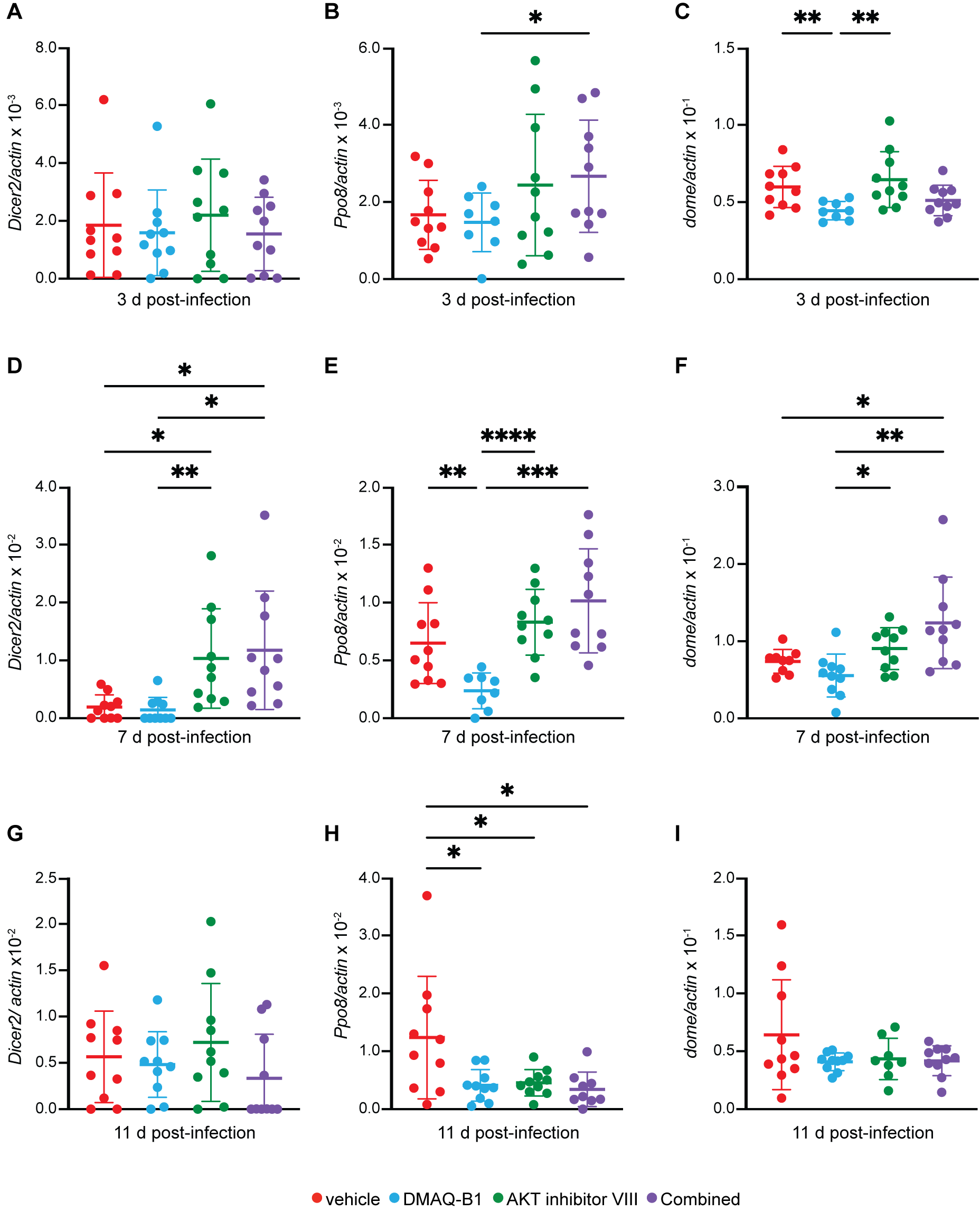

Supplement: S5 Fig — Induction of additional immune genes at (A-C) 3, (D-F) 7, and (G-I) 11 d p.i. in adult female Ae. aegypti was measured by qRT-PCR. RNAi associated genes (A, D, G) Dicer2 and (B, E, H) Ppo8 and JAK/STAT (C, F, I) dome were measured. (*p < 0.05; **p<0.01; ***p<0.001; **** p<0.0001, unpaired t test with Welch’s correction for multiple comparisons). Closed circles represent individual replicates. Outliers were identified using a ROUT test (Q = 5%) and removed. Horizontal bars represent mean and error bars represent SD. Results represent duplicate independent experiments. (TIF) [file ppat.1010411.s007.tif]

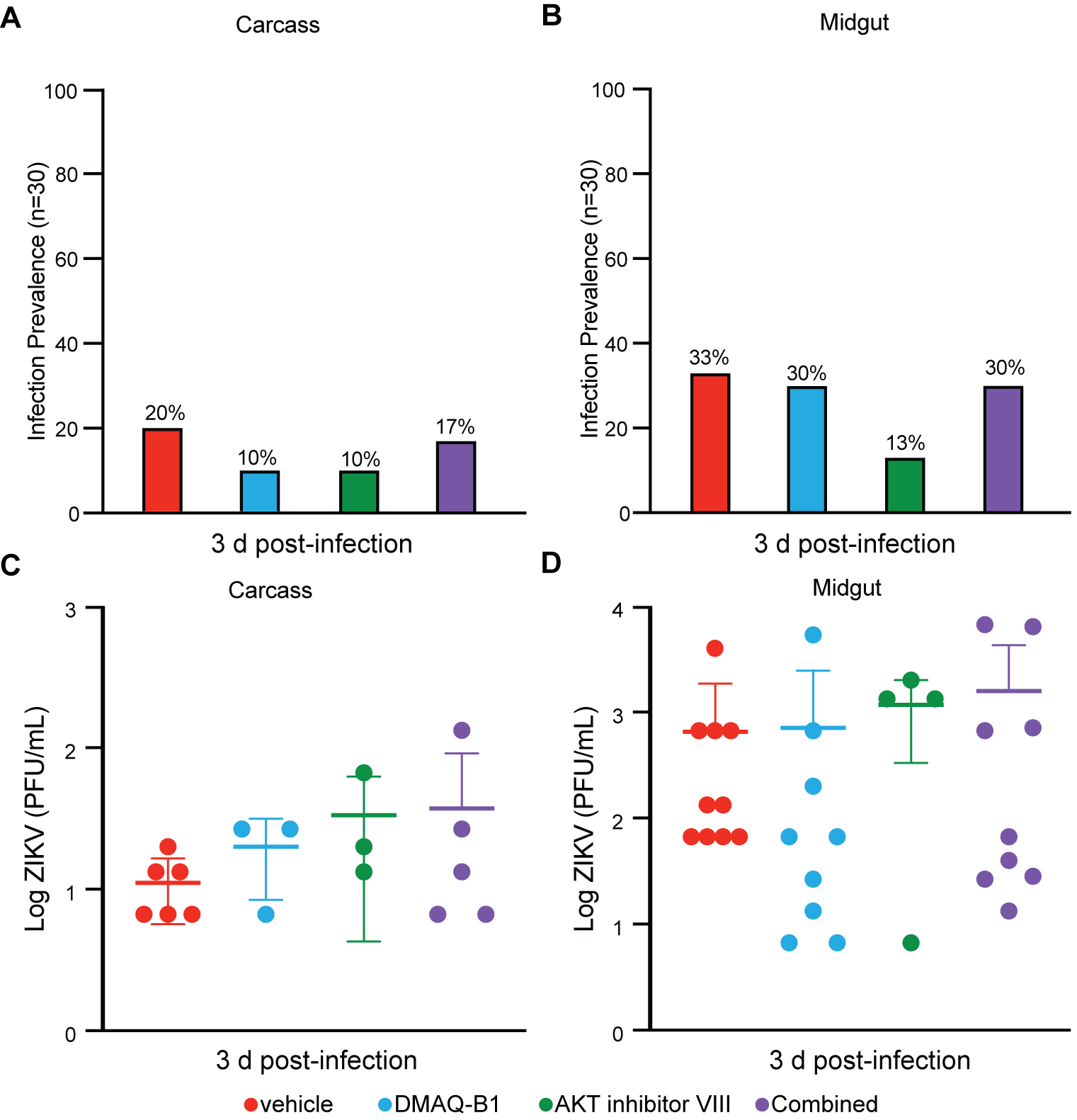

Supplement: S6 Fig — Ae. aegypti were primed with 1% DMSO, 10 μM DMAQ-B1, 10 μM AKT inhibitor VIII, or combined drugs and infected with ZIKV by bloodmeal. Mosquitoes (n = 30) were collected at 3 d p.i. and individual midguts, pairs of salivary glands, and carcasses were prepared and titered by standard plaque assay. Infection prevalence was determined by comparing the number of mosquitoes with detectable virus to the total mosquitoes in the sample. Viral titer was measured in mosquitoes that were positive for ZIKV. There were no differences in infection prevalence or viral titers among conditions. Open circles represent biological replicates. Outliers were identified using a ROUT test (Q = 5%) and removed. Bars represent the mean. Error bars represent SDs. Data are representative of duplicate independent experiments. (TIF) [file ppat.1010411.s008.tif]
