# Supplementary material for: Coupled small molecules target RNA interference and JAK/STAT signaling to reduce Zika virus infection in Aedes aegypti
Source: PLoS Pathog. 2022 Apr 4;18(4):e1010411. doi: 10.1371/journal.ppat.1010411 (PMC9017935; doi:10.1371/journal.ppat.1010411)
Supplement: Attachment — Submitted filename: PlosPath_Rebuttal-Letter_Final.docx [file ppat.1010411.s009.docx]

23 February 2022

RE: PPATHOGENS-D-21-01874

"Coupled small molecules target RNA interference and JAK/STAT signaling to reduce Zika virus infection in *Aedes aegypti*"

Dear Dr. McGraw and the Editors of *PLOS Pathogens*,

Thank you for the opportunity to submit our revised manuscript (PPATHOGENS-D-21-01874), titled “Coupled small molecules target RNA interference and JAK/STAT signaling to reduce Zika virus infection in *Aedes aegypti*.” We are grateful to the expert reviewers who critiqued our manuscript. We have taken all the recommendations into consideration and have written a point-by-point response indicating how we addressed each reviewer comments or concerns. We believe that the additional experiments, revised analyses, and modifications of the text have substantially improved our manuscript.

We were encouraged by the expert reviewers’ positive comments. Reviewer #1 wrote that our study was “…well-conducted, utilizing appropriate controls and thoughtfully designed while the results are quite promising and are a clear advancement to the field.” Reviewer #2 stated that our manuscript “should be of interest to readers of PLOS Pathogens.” Both Reviewer #1 and Reviewer #2 provided constructive comments that will greatly enhance the manuscript to the quality expected for publication at *PLOS Pathogens*.

Along with this letter, we provide point-by-point responses to the queries raised by the reviewers, which are written in below in italics. In the edited text, all written revisions are indicated with red, underlined font. Line numbers cited in the responses to the reviewers’ comments correspond to the line numbers in the marked-up text document. We hope that upon reviewing our improved manuscript you and the editorial team will agree that this manuscript would be a valuable contribution to your journal.

Reviewer 1:

**PART 1**

1. *“In “Coupled small molecules target RNA interference and JAK/STAT signaling to reduce Zika virus infection in Aedes aegypti”, Trammell and colleagues utilized in vitro and in vivo models to advance the understanding of ingested vertebrate factors of the insulin/insulin-like growth factor signaling cascade could simultaneously induce both the JAK/STAT and RNAi antiviral pathways and decrease infection of Aedes aegypti by Zika virus (ZIKV). Given the dearth of vaccines and therapeutics, the only methods for controlling most arbovirus outbreaks are avoidance and abatement of mosquitoes, and use of insecticides. Therefore, there is an enormous value in methods of modulating mosquito infectability by arboviruses.”*

*The work presented in well-conducted, utilizing appropriate controls and thoughtfully designed while the results are quite promising and are a clear advancement to the field. Despite this however, there is some concern in the design of the in vivo element of the experiment, that is elaborated on specifically below. In addition, minor grammatical and writing suggestions are noted.”*

We thank Reviewer 1 for their feedback and positive assessment of our manuscript. We are excited to hear that our results are promising and add valuable information to the field. We appreciate missed grammatical and writing hiccups and hope this resubmission addresses these concerns. To address the design of our *in vivo* element, and in turn enhance the translatability of our findings, we have added an additional experiment in which mosquitoes were administered our proposed small molecules in a manner similar to how established bait-traps are used in the field. Our findings of this additional experiment should address concerns regarding the applicability of our proposed approach and efficacy long-term.

**PART 2**

1. *“While the in vitro data is largely complete, there is some concern about the design of the mosquito experiment. Specifically, the two small molecules were provided to mosquitoes within the bloodmeal itself alongside the virus. Given that the authors note the real-world application of this mechanism as a field-based strategy, such an experimental paradigm is quite unrealistic. This is because mosquitoes would likely encounter the bait stations/lures impregnated with the compounds either before or after ingesting a virus laden bloodmeal. It would therefore be advantageous to present a more field accurate mosquito experiment alongside the experiment currently depicted in Figure 3.”*

We agree that our argument regarding the translatability and real-world application of our findings can be improved. The initial goal of this study was to establish and evaluate the mechanism of action that our small molecules possess and the antiviral effect they have against Zika virus *in vitro* and *in vivo*. However, our findings and applicability can be further enhanced by applying our findings into established field methods like bait stations.

To address this, we conducted survival and qRT-PCR analysis of female *Aedes aegypti* mosquitoes that are continuously exposed to our small molecule treatment conditions via a sucrose water source. Specifically, 6-9 day old mosquitoes were collected and maintained on small-molecule supplemented-sucrose water over a 14 day period. We measured mosquito survival during this period and collected whole mosquitoes at 3, 7, and 11 days to measure induction of RNAi- and JAK/STAT-related genes, in the absence of virus, using qRT-PCR. The purpose of this experiment was to: 1) evaluate the potential of our small molecules as a means to prime RNAi and JAK/STAT pathways independently of Zika infection, and 2) identify potential toxicity of continuous small molecule exposure as opposed to a single treatment as previously described. We demonstrate that not only is there no significant difference in survival among vehicle-, single-, or combined drug-treated mosquitoes, but we observed a similar gene induction profile as mosquitoes that received a single treatment via bloodmeal. We observed induction of both RNAi and JAK/STAT up to 7 days and loss of signaling at 11 days.

These additions are presented in the revised manuscript as:

- “Figure 2: Continuous drug treatment via sucrose and water results in induction of RNAi and JAK/STAT signaling in *Aedes aegypti* mosquitoes.”
- “Figure S2: Continuous drug treatment via sucrose and water does not impact mosquito survival.”
- *“DMAQ-B1- and AKT inhibitor VIII-supplemented sucrose water induces activation of RNAi and JAK/STAT signaling in* Aedes aegypti

To model established sugar-bait strategies implemented in the field (13) and translatability of our *in vitro* findings, we next evaluated the effect DMAQ-B1 and AKT inhibitor VIII treatment possesses against IIS-dependent antiviral response in adult female *Ae. aegypti*. Aged-matched 6-9-day old female mosquitoes were continuously exposed to 10% sucrose water supplemented with either 1% DMSO vehicle control, individual, or combined 10 μM DMAQ-B1 and 10 μM AKT inhibitor VIII. Survival was measured over a 14-day treatment period with no difference between treatment conditions (**Fig. S2**). Whole mosquitoes were collected at 3 (**Fig. 2A-D**), 7 (**Fig. 2E-H**), and 11 days (**Fig. 2I-L**) for analysis of RNAi and JAK/STAT gene induction by qRT-PCR. *AGO2* and *p400* were examined as markers of RNAi (**Figs. 2A-B, 2E-F, 2I-J**) (30,31), while *Vago2* and *vir-1* were examined as downstream effectors of JAK/STAT (**Figs. 2C-D, 2G-H, 2K-L**) (32,33). At day 3 we observed higher expression of RNAi-related genes in combined drug-treated mosquitoes compared to our vehicle control (**Fig. 2A-B**) but no difference in JAK/STAT-related genes (**Fig. 2C-D**). At day 7, however, we saw that RNAi and JAK/STAT gene induction were significantly higher in our combined drug-treated mosquitoes compared to our vehicle control (**Fig. 2E-H**). By day 11 we observed a loss in immune gene induction in the combined drug-treated mosquitoes (**Fig. 2I-L**). This leads us to infer that the effectiveness and activity of DMAQ-B1 and AKT inhibitor VIII, at least in combination, induces RNAi and JAK/STAT genes up to 7 days of continuous treatment but gene induction is reduced by 11 days, possibly due to negative feedback that inhibits the induction of these genes during continuous feeding. Because of this limited toxicity, transcript profile, and range of efficacy, we conclude that DMAQ-B1 and AKT inhibitor VIII induce RNAi and JAK/STAT signaling in a similar manner as we observed *in vitro*.” **(Lines 176-200)**

- “We have demonstrated that exposure of DMAQ-B1 and AKT inhibitor VIII through either continuous exposure (**Fig. 2**) or a single treatment (**Fig. 3**) was sufficient to induce activation of both antiviral pathways independently of ZIKV infection. More importantly, even with the defined time period during which these drugs appear to induce pathway activation, there was still a significant reduction in ZIKV positive- and vector competent-mosquitoes following IIS pathway activation (**Fig. 4**).” **(Lines 377-382)**
- “Toxicity was evaluated similarly in 6-9 day old female mosquitoes treated by either drug-treated sucrose water or drug-treated bloodmeal. Sucrose water-treated mosquitoes were provided 10% sucrose water with either 10 μM DMAQ-B1, 10 μM AKT VII inhibitor, combined treatment of both 10 μM DMAQ-B1 and 10 μM AKT inhibitor, or 1% DMSO vehicle control *ad libitum*. Mosquitoes were maintained for 14 d to monitor mortality.” **(Lines 457-462)**

2. “*The authors have framed the application of this study to mosquito control via bait stations, however in “Dong S, Dimopoulos G. Antiviral Compounds for Blocking Arboviral Transmission in Mosquitoes. Viruses. 2021 Jan;13(1):108.” it was noted that the attractive toxic sugar bait strategy “also attracted and killed nontargeted beneficial insects such as honeybees and parasitoid wasps”. A previous study “Ahlers LRH, Trammell CE, Carrell GF, Mackinnon S, Torrevillas BK, Chow CY, et al. Insulin Potentiates JAK/STAT Signaling to Broadly Inhibit Flavivirus Replication in Insect Vectors. Cell Rep. 2019 Nov;29(7):1946-1960.e5” demonstrated that the Drosophila melanogaster are also affected by insulin. While the compounds in the present study do not appear toxic at the concentrations tested, it is reasonable that these concentrations would also have consequences to other insects that sugar feed. Is there any evidence that these agents would have pleiotropic effects in non-target species causing changes to longevity, fecundity, etc? While follow-up experiments to address this are not within the purview of this paper, this limitation and gap should not be ignored in the discussion.”*

We agree with reviewer 1 in that while these pleiotropic effects are beyond the scope of this paper, it is important to discuss these limitations and gaps in knowledge to effectively discuss the potential our finds have in being translated into a field setting.

These additions are presented in the revised manuscript as:

- “While this broad-antiviral effect is promising, further investigation is necessary to elucidate the full impact our proposed small molecules may have when introduced as a field-based strategy. Because of the limited studies on the effects of DMAQ-B1 and AKT inhibitor VIII on insects, it is unknown if these drugs may be hazardous to beneficial species that could be exposed during mosquito treatment (13,45).” **(Lines 388-392)**

**PART 3**

1. *“Line 38: The word “memetic” is incorrect. The correct word is “mimetic” which the authors use throughout the manuscript.”*

We thank the reviewer for pointing out this mistake. We have corrected this to use “mimetic” to be consistent with the rest of the manuscript.

2. *“Line 49: The statement “particularly those transmitted by mosquitoes” is unnecessary.”*

We thank the reviewer for raising this concern. We originally included this statement to clarify that our focus is on mosquito-borne arboviruses and not those transmitted via ticks or flies. We recognize that arboviruses, regardless of the vector, pose a significant threat globally and thus have removed that statement from the updated manuscript.

3. *“Line 67: The use of the phrase “virus-free” regions is incorrect.”*

We acknowledge that the use of this phrase may be misleading.

These additions are presented in the revised manuscript as:

- “Global warming and urbanization have permitted mosquitoes and arboviruses to spread into regions that previously lacked mosquito infection and mosquito-to-human virus transmission (1,2).” **(Lines 68-70)**

4. *“Figure 3: There appears to be mismatch between mosquito populations between prevalence and titers. In all prevalence figures (3A-C, 3G-I), 30 individual mosquitoes were screened for presence of virus. For an example, consider the vehicle condition in 3A and 3D. Figure 3A shows 33% of mosquitoes were infected with ZIKV which corresponds to 10 mosquitoes. The corresponding dot plot in D has 9 dots. This appears to be a common occurrence throughout Fig 3. Can this be clarified?”*

We thank the reviewer for identifying this issue and giving us the opportunity to clarify. As we state in our “Quantification and Statistical Analysis” section, data were analyzed to remove outliers by ROUT test (Q=5%) to variability between individual samples. We acknowledge that this should also be restated in the figure captions.

These additions are presented in the revised manuscript as:

- “Outliers were identified using a ROUT test (Q=5%) and removed.” **(Lines 787-788)**
- “Outliers were identified using a ROUT test (Q=5%) and removed.” **(Line 798)**
- “Outliers were identified using a ROUT test (Q=5%) and removed.” **(Lines 810-811)**
- “Outliers were identified using a ROUT test (Q=5%) and removed.” **(Lines 825-832)**
- “Outliers were identified using a ROUT test (Q=5%) and removed.” **(Line 881)**
- “Outliers were identified using a ROUT test (Q=5%) and removed.” **(Line 895)**
- “Outliers were identified using a ROUT test (Q=5%) and removed.” **(Lines 906-907)**

5. *“Lines 237-239: The phrase “…the need for host-virus interactions research” reads awkwardly. Consider rephrasing.”*

We agree that the phrasing of this statement should be improved.

The manuscript has been revised as:

- “Recent ZIKV epidemics have demonstrated the need for research focused on identifying novel and more effective targets at the vector level.” **(Lines 353-354)**

6. *Line 242: The word “therapeutic” in entirely inappropriate here.*

We acknowledge that the use of the word “therapeutic” can be construed as misleading or inaccurate given the context in which these small molecules were used. We have removed the word in that statement.

7. *Line 277-278: Clarify means by which mosquitoes were given access to sugar and water.*

We added additional information regarding how mosquitoes had access to sucrose and water.

The statement now reads as:

- “Adult mosquitoes were provided continuous access to water and 10% sucrose *ad libitum* using soaked cotton balls that were replaced every other day prior to and following bloodmeal feeding. Females were allowed to feed on defibrinated sheep blood (Colorado Serum Company 31123) supplemented with 1mM ATP using an artificial feeding system to stimulate oogenesis.” **(Lines 414-418)**

Reviewer 2:

**PART 1**

1. *“The present paper titled ‘Coupled small molecules target RNA interference and JAK/STAT signaling to reduce Zika virus infection in Aedes aegypti’ by Trammell et al., describes the antiviral properties of two small molecules which induce the RNAi and Jak/STAT pathways. The authors use mostly qRT-PCR to detect RNAi and Jak/STAT pathway gene expression to determine the impact of drug treatment on these pathways and virus plaque assay to detect the impact on virus replication. The major conclusions that the authors make are that a combination of two drugs (which feed into insulin signaling) can increase antiviral responses, specifically RNAi and Jak/STAT, and reduce virus transmission in vivo.*

*The paper is generally well-written and organized. The topic of study should be of interest to readers of PLOS Pathogens, but this reviewer has some concerns with the clarity of the data and whether the conclusions are supported.”*

We thank Reviewer 2 for their feedback and support regarding the organization and interest our presented manuscript has for readers of *PLOS Pathogens*. We hope that the addition of proposed experiments into the new manuscript and within this rebuttal will clarify our interpretation of results and solidify support in the publication of this manuscript.

**PART 2**

1. *“My major concerns are with the distinction of the two pathways (RNAi and JAK/STAT) and what the data is really supporting (see specific issues below). Some experiments indicate a much more complicated overlap between the results of the two drugs used, and Figure 4C, which would be a crucial experiment to identify the mechanism of these drugs, does not clarify but rather confuse interpretation of results. While I think the antiviral effect of the two drugs is undeniable and important, this paper is aiming at a clear mechanistic level of how these drugs work in the context of virus infection, but it is not delivering on that. As I said, my main worry comes from the questions that Figure 4C brings up (see more specific comments below). In addition, there are other functional outputs for RNAi (such as small RNA sequencing) that could have been considered to support that part of the mechanism.”*

*“I am not expecting all of the experiments mentioned below, but a set of these could strengthen this paper on the mechanistic side. I think right now the mechanism is not clear and one or two of these experiments should be considered (whichever experiments provide useful/clarifying data):*

***- repetition of the experiment(s) in Figure 4C***

***- verification of knockdown in these samples at the time when virus is measured***

***- quantification of other RNAi and Jak/STAT genes***

*- knockdown of other RNAi and JAK/STAT genes and addition to Fig 4C*

***-*** ***mimicking the proposed function of the drug through other experiments to indicate whether the drugs are antiviral through the proposed mechanism***

*- other readouts to test any impact on RNAi, such as small RNA sequencing (but there may be other ways to test this)”*

We thank the reviewer for their support. While the antiviral effect and significance as a potential control strategy in the field, our overall investigation into the mechanism of action of our small molecules can be further evaluated and supported.

We also thank the reviewer for their flexibility in suggested experiments to further support our proposed mechanism of action. We performed the experiments in bold above as suggested by the Reviewer. First, we repeated our experiment in Figure 4C (now 5D in the updated manuscript) and updated the figure by showing the pooled data from the triplicate experiments. As shown in Figure R1, we demonstrate that our findings are reproducible and show a consistent difference in siControl and the individual and stacked siRNA treated cells.

**Figure R1: Individual and pooled replicates of ZIKV titer analysis of drug-treated siControl (siCt), siAGO2, siVir-1, and siAGO2+siVir-1.** (A-C) ZIKV titer of each individual replicate of drug-treated Aag2 cells following siRNA transfection. Cells were treated for 24 h following 48 h siRNA transfection and then infected with ZIKV (MOI 0.01). Supernatant was collected and titered at 2 d p.i.. Each individual replicate is (D) pooled and presented as Figure 5D. (*p<0.05; ***p<0.001 **** p<0.0001,Two-way ANOVA with uncorrected Fisher’s LSD test). Closed circles represent individual well replicates.

We have also included analysis comparing within treatment conditions and demonstrate a significant difference between vehicle treated cells that received siControl, siVir-1, and siAGO2+siVir-1 transfection. This additional statistical analysis is compiled and included in an excel spreadsheet to list out the multiple comparisons completed by two-way ANOVA (**Table S2**). A major finding of our study is the comparison between the siControl group to the individual and double siRNA-transfected cells to evaluate whether we lose our drug-mediated antiviral effect in the absence of functional RNAi and JAK/STAT signaling as we previously established in **Figure 1K**. There is a significant difference and loss of antiviral protection in our siAGO2, siVir-1 and siAGO2+siVir-1 transfected cells (**Figure 5D, Table S2**). We also demonstrated a difference in titer in vehicle-treated cells when comparing siCt to siVir-1 and siAGO2+siVir-1 but not siAGO2 (**Table S2**). In addition, we measured expression of *AGO2* and *vir-1* levels in our transfected cells at 72h and 120h post transfection with removal of siRNA at 48 h. These timepoints correspond to time of ZIKV infection and time of collection for viral titer, respectively. We determined that while *vir-1* expression remained inhibited up to time of supernatant collection, *AGO2* levels were restored between time of infection and time of collection. This could cause the lack of a significant increase in ZIKV titer between vehicle-treated cells in the siAGO2 group compared to siCt since RNAi could become responsive during the infection time course. We have updated **Figures 5B, 5C** to include this gene expression. We also added **Figure 5A** to provide a schematic regarding experimental design and rationale for timepoints measured.

These additions are presented in the revised manuscript as:

- “We measured *AGO2* and *vir-1* expression at 48, 72, and 120 h in accordance with siRNA removal, when cells were infected, and when supernatant was collected, respectively (**Fig. 5A**). We observed significantly reduced gene expression at 48 h and 72 h post transfection for both individual and stacked siRNA treatments compared to cells that were treated with control, non-targeting siRNAs (42). Individual siAGO2 treated cells exhibited a 68% and 41% reduction of AGO2 expression at 48 h and 72 h, respectively, whereas stacked siRNA treated cells had a 84% and 99% reduction at the same timepoints (**Fig. 5B**). We observed that *AGO2* expression returned to control levels in our siAGO2 and siAGO2 + siVir-1 transfected cells by 120 h. Expression levels of *vir-1* in siVir-1- and stacked transfected cells were reduced at all timepoints measured (**Fig. 5C**). In siVir-1-transfected cells, *vir-1* expression was reduced by 69% at 48 h, 88% at 72 h, and 74% at 120 h. Stacked-transfected cells had reduced *vir-1* expression at 82% at 48 h, 77% at 72 h, and 63% at 120 h.” **(Lines 299-311)**
- “Interestingly, while we observed a loss in antiviral protection when comparing all data among gene knockdown groups (**Table S2, Sheet1**), we observed a significant increase in viral titers only among the vehicle control groups when comparing siControl to siVir-1 or combined knockdown (**Table S2, Sheet2**). One explanation for why we did not observe differences between the siControl and siAGO2 group during vehicle treatment may be that while AGO2/RNAi is inhibited at the time of infection, its activity is restored by the time of sample collection (**Fig. 5B**).” (**Lines 317-340**)
- “(A) Experimental schematic illustrating process in which cells are transfected with siRNA for 48 h, drug-treated for 24 h prior to infection (72 h), and viral titer measured at 2 d p.i. (120 h).” (**Lines 815-817**)
- “Results are presented as pooled data of triplicate, independent experiments.” (**Lines 832-833**)

To further demonstrate the validity of our proposed mechanism, we measured induction of other RNAi and JAK/STAT genes within our ZIKV-infected mosquitoes as suggested. These data are presented in **Figure S5** and **Table S1**. We demonstrate that additional RNAi and JAK/STAT genes (*Dicer2*, *Ppo8*, and *dome*) are partially induced at 3 d p.i., fully induced within our combine treated mosquitoes at 7 d p.i., and reduced by 11 d p.i.. This is a similar trend we observed with other genes tested in **Figure 3**. We hope that this helps support our claim that combined drug treatment induces various components of RNAi and JAK/STAT.

These additions are presented in the revised manuscript as:

- “Expression levels of other related RNAi genes including *Dicer2* and *Ppo8* (30,37) and JAK/STAT *dome* (38) were enhanced in combined drug-treated mosquitoes 7 d p.i. (**Fig. S5**), which affirms the effect that DMAQ-B1 and AKT inhibitor VIII have for RNAi and JAK/STAT signaling in *Ae. aegypti* mosquitoes.” (**Lines 235-238**)
- “Figure S5: Additional RNAi and JAK/STAT genes were induced in small molecule treated mosquitoes at 3, 7, and 11 d p.i. Induction of additional immune genes at (A-C) 3, (D-F) 7, and (G-I) 11 d p.i. in adult female *Ae. aegypti* was measured by qRT-PCR. RNAi associated genes (A, D, G) *Dicer2* and (B, E, H) *Ppo8* and JAK/STAT (C, F, I) *dome* were measured. (*p < 0.05; **p<0.01; ***p<0.001; **** p<0.0001, unpaired t test with Welch’s correction for multiple comparisons). Closed circles represent individual replicates. Outliers were identified using a ROUT test (Q=5%) and removed. Horizontal bars represent mean and error bars represent SD. Results represent duplicate independent experiments.” (**Lines 885-896**)

Finally, we evaluated the effect that inhibiting PI3K, the kinase that phosphorylates Akt, has on our observed antiviral effect. To achieve this, we conducted the same experimental design as presented in **Figure 1J** with the substitution of AKT inhibitor VIII with 10 μM LY294002, a potent inhibitor of PI3K phosphorylation. Aag2 cells were primed with either vehicle, individual, or combined drug treatment and then infected with ZIKV (MOI 0.01 PFU/cell) as previously described. Supernatant was collected at 1- and 3-days post infection to measure ZIKV levels within each treatment condition. In **Figure R2**, we demonstrate that while some level of antiviral protection was observed in our individual and combined LY294002 treated cells, it is not to the same degree as AKT inhibitor VIII. We propose this is because the inhibition of PI3K may also inhibit DMAQ-B1-mediated activation of ERK’s downstream targets, thus partially negating the overall antiviral effect during combined drug treatment.

**Figure R2:** Aag2 cells received vehicle, 1 μM DMAQ-B1, 10 μM LY294002, or combined drug treatment for 24 h were infected with ZIKV (MOI 0.01 PFU/cell) and supernatant was collected at 1 and 3 d p.i. Supernatant virus was titered by standard plaque assay (***p<0.001; ****p<0.0001, Two-way ANOVA with Tukey’s correction). Closed circles represent individual well replicates. Results are representative of triplicate independent experiments.

**PART 3:**

1. *“Figure 4C is highly confusing – not in its design, but the results are confusing. While results for the drug treatments look as expected in the control siRNA samples, it is highly surprising that essentially all other siRNA/drug combinations look identical in ZIKV titers. This result removes the clear explanations for how RNAi and JAK/STAT may be induced by the different drugs. For example, in Figure 1I, the authors show no significant impact of DMAQ-B1 treatment on vir-1 expression, but in Figure 4C, vir-1 silencing removed any effect that DMAQ-B1 had on ZIKV titers – so the effect was dependent on vir-1? And if the impact is so clearly vir-1 dependent, how can it be just as clearly Ago-2 dependent? Essentially, there should be more defined differences between the drug/siRNA combinations to support the model shown in Figure 4D. This figure legend also provides no information on independent replicates or even experimental replicates and this reviewer is a bit concerned about the validity of these results. Maybe some independent replicate experiments are needed to try to understand what is going on? Expression of vir-1 and Ago-2 in these exact samples at the time when virus was harvested also would have been useful (as opposed to just at 48h post transfection).”*

We thank the reviewer for their in depth critique and analysis of Figure 4 (now Figure 5) in order for us to present our findings in the most effective and clear way as possible. We hope that we have addressed and corrected these concerns as previously described in Part 2 and the presentation of **Table S2** and **Figure R1**.

2. *Another larger concern is the difference in this experimental design to the use of sugar-bait feeding. While I understand that this is an initial study, the authors imply that these small molecules could be sugar-fed to mosquitoes and reduce transmission – sugar feeding can change how/when/how efficiently these drugs are taken up and it would have been nice to see an experiment where mosquitoes are provided a virus containing blood-meal while being held on sugar-water (or other sugar bait) that contains the drugs – the potential constant low-level exposure with targeting to the crop instead of the midgut could impact results in either direction.*

We thank the reviewer for recognizing that this was intended as an initial, proof of concept study, but agree that further investigation regarding the effect that our small molecules possess when introduced continuously via water and sucrose, similar to how bait traps are used in the field, would greatly enhance the applicability and translatability of our findings.

To address this concern that was also brought up by Reviewer 1, we conducted survival and qRT-PCR analysis of female *Ae. aegypti* mosquitoes that are continuously exposed to our small molecule treatment conditions in both water and sucrose sources. Specifically, 6-9 day old mosquitoes were collected and maintained on small-molecule supplemented-water and sucrose over a 14 day period. We measured mosquito survival over 14 days and collected whole mosquitoes at 3, 7, and 11 days to measure induction of RNAi and JAK/STAT-related genes, in the absence of virus, by qRT-PCR. The purpose of this experiment was: 1) evaluate the potential of our small molecules to prime RNAi and JAK/STAT pathways independently of Zika infection and 2) identify potential toxicity of continuous small molecule exposure as opposed to a single treatment as previously described. We demonstrate that not only is there no significant difference in survival between vehicle and single- and combined-treated mosquitoes, but we observed a similar gene induction profile as mosquitoes that received a single treatment via bloodmeal. We observed induction of both RNAi and JAK/STAT up to 7 days and loss of signaling at 11 days.

These additions are presented in the revised manuscript as:

- “Figure 2: Continuous drug treatment via sucrose and water results in induction of RNAi and JAK/STAT signaling in *Aedes aegypti* mosquitoes.”
- “Figure S2: Continuous drug treatment via sucrose and water does not impact mosquito survival.”
- *“DMAQ-B1- and AKT inhibitor VIII-supplemented sucrose water induces activation of RNAi and JAK/STAT signaling in* Aedes aegypti

To model established sugar-bait strategies implemented in the field (13) and translatability of our *in vitro* findings, we next evaluated the effect DMAQ-B1 and AKT inhibitor VIII treatment possesses against IIS-dependent antiviral response in adult female *Ae. aegypti*. Aged-matched 6-9-day old female mosquitoes were continuously exposed to 10% sucrose water supplemented with either 1% DMSO vehicle control, individual, or combined 10 μM DMAQ-B1 and 10 μM AKT inhibitor VIII. Survival was measured over a 14-day treatment period with no difference between treatment conditions (**Fig. S2**). Whole mosquitoes were collected at 3 (**Fig. 2A-D**), 7 (**Fig. 2E-H**), and 11 days (**Fig. 2I-L**) for analysis of RNAi and JAK/STAT gene induction by qRT-PCR. *AGO2* and *p400* were examined as markers of RNAi (**Figs. 2A-B, 2E-F, 2I-J**) (30,31), while *Vago2* and *vir-1* were examined as downstream effectors of JAK/STAT (**Figs. 2C-D, 2G-H, 2K-L**) (32,33). At day 3 we observed higher expression of RNAi-related genes in combined drug-treated mosquitoes compared to our vehicle control (**Fig. 2A-B**) but no difference in JAK/STAT-related genes (**Fig. 2C-D**). At day 7, however, we saw that RNAi and JAK/STAT gene induction were significantly higher in our combined drug-treated mosquitoes compared to our vehicle control (**Fig. 2E-H**). By day 11 we observed a loss in immune gene induction in the combined drug-treated mosquitoes (**Fig. 2I-L**). This leads us to infer that the effectiveness and activity of DMAQ-B1 and AKT inhibitor VIII, at least in combination, induces RNAi and JAK/STAT genes up to 7 days of continuous treatment but gene induction is reduced by 11 days, possibly due to negative feedback that inhibits the induction of these genes during continuous feeding. Because of this limited toxicity, transcript profile, and range of efficacy, we conclude that DMAQ-B1 and AKT inhibitor VIII induce RNAi and JAK/STAT signaling in a similar manner as we observed *in vitro*.” **(Lines 176-200)**

- “We have demonstrated that exposure of DMAQ-B1 and AKT inhibitor VIII through either continuous exposure (**Fig. 2**) or a single treatment (**Fig. 3**) was sufficient to induce activation of both antiviral pathways independently of ZIKV infection. More importantly, even with the defined time period during which these drugs appear to induce pathway activation, there was still a significant reduction in ZIKV positive- and vector competent-mosquitoes following IIS pathway activation (**Fig. 4**).” **(Lines 377-382)**
- “Toxicity was evaluated similarly in 6-9 day old female mosquitoes treated by either drug-treated sucrose water or drug-treated bloodmeal. Sucrose water-treated mosquitoes were provided 10% sucrose water with either 10 μM DMAQ-B1, 10 μM AKT VII inhibitor, combined treatment of both 10 μM DMAQ-B1 and 10 μM AKT inhibitor, or 1% DMSO vehicle control *ad libitum*. Mosquitoes were maintained for 14 d to monitor mortality.” **(Lines 457-462)**

3. *Line 193: For Figure 3D-F, the authors state that viral titers were reduced less than 2-fold, however based on the y-axis, the data is shown in a logarithmic scale and was reduced by more than 10-fold in the midguts. Carcass data was unclear/not significant, and the salivary gland data probably had too few data points. I think it might be useful to not condense these descriptions into one sentence, but clearly describe each figure panel. For 11d p.i., the authors also just say that it is more than two-fold when it is really more than 10-fold (unless I am misinterpreting something).*

We thank the reviewer to identifying this discrepancy and have made the appropriate changes to describe the fold changes more accurately.

We have added the following statements:

- “At 7 d p.i. infection prevalence was significantly reduced midgut and salivary gland tissues but not carcasses from combined drug-treated mosquitoes (**Figs. 4A-C**). The titer of ZIKV-positive mosquitoes at 7 d p.i., however, was only reduced in the midguts of combined drug-treated mosquitoes with an approximate 30-fold reduction. There was no difference in carcass and salivary gland titers (**Figs. 4D-F**). By 11 d p.i., both infection prevalence (**Figs. 4G-I**) and viral titer (**Figs. 4J-L**) were significantly reduced relative to vehicle control in mosquitoes treated with individual and combined drug treatment across tissue types. Titers in ZIKV-positive mosquitoes were reduced by ~42-fold in carcass, ~15-fold in midgut, and ~24-fold in salivary glands (**Figs. 4J-L**).” (**Lines 247-261**)

4. *Line 198-200: The authors compare their data to another study in which 4.8log10 PFU/mL was determined as the required titer in salivary glands to transmit ZIKV. I think the authors may need to be careful with these numbers and the subsequent sentence about ‘reducing numbers below the level of transmission’ – the reason is that all other salivary glands from control mosquitoes also had titers below this 4.8log10 PFU/mL threshold, indicating that either the methods were not as sensitive here as in the other study, or that none of these mosquitoes were transmission competent (in which case there was no reduction below the threshold since none of the mosquitoes passed it). It does not take away from these impressive results, but the wording should be adjusted a bit in my opinion.*

*The discussion uses and mentions many of my points above as well and would need some adjustment according to my concerns (e.g. the reduction below transmission potential in line 251, or the proposed sugar-bait feeding that was not addressed here experimentally).*

We appreciate that our wording and interpretation could be misconstrued. We have elected to remove this statement and interpretation. Based on the reductions in ZIKV-positive mosquitoes and titers in positive mosquitoes, we have clarified that these mosquitoes would have a reduced capacity to transmit in subsequent bloodmeals.

We have added the following statements:

- “Mosquitoes that received the combined drug treatment were not only less likely to be ZIKV-positive, but salivary gland viral load was also substantially reduced in positive mosquitoes. These observations suggested that combined drug treatment and coordinated activation of RNAi and JAK/STAT provides antiviral immunity against ZIKV that effectively reduced infection prevalence and viral load compared to vehicle controls.” (**Lines 263-268**)
- “By targeting IIS as a mediator of two antiviral pathways, we reduced both infection prevalence and virus titers, outcomes predicted to reduce the likelihood of transmission.” (**Lines 372-374**)
- “More importantly, even with the limited time range in which these drugs appear to induce pathway activation, there was a significant reduction in ZIKV positive- and vector competent- mosquitoes following IIS pathway activation (Fig. 4).” (**Lines 379-382**)

5. *Normalizing the qRT-PCR data to the control would help a bit with interpretation, especially in Figure 4A, were the knockdown of gene expression is shown. By normalizing these (ΔΔct or other fold normalization), interpretation would be more intuitive.*

We recognize that normalizing the qRT-PCR data would help within general interpretation, but we elected to normalize to *actin* so that data for the control group is also shown rather than normalized to 1. Nevertheless, we have included fold changes in the text important comparisons of data shown in Figures 5B and 5C.

We have added the following statements for clarity:

- “We observed significantly reduced gene expression at 48 h and 72 h post transfection for both individual and stacked siRNA treatments compared to cells that were treated with non-targeting siRNAs as a control (42). Individual siAGO2 treated cells exhibited 68% and 41% reductions in *AGO2* expression at 48 h and 72 h, respectively, whereas stacked siRNA treated cells exhibited 84% and 99% reductions at the same timepoints (**Fig. 5B**). We observed that *AGO2* expression returned to control levels in our siAGO2 and siAGO2 + siVir-1 transfected cells by 120 h. Expression of *vir-1* in siVir-1- and stacked transfected cells was reduced at all timepoints measured (**Fig. 5C**). In siVir-1-transfected cells, *vir-1* expression was reduced by 69% at 48 h, 88% at 72 h, and 74% at 120 h. In stacked-transfected cells, *vir-1* expression was reduced by 82% at 48 h, 77% at 72 h, and 63% at 120 h.” (**Lines 301-311**).
- “Expression of *Ae. aegypti AGO2, p400, Vago2*, and *vir-1* were measured using SYBR Green reagents (ThermoFisher K0222) and normalized to actin to measure endogenous gene levels for all treatment conditions.” (**Lines 502-504)**

6. *Figure 1H-I: The label on the x-axis says 1 day post infection, but the text and the figure legend do not indicate that these cells were infected. Either the next/legend neglected to say that ago2 and vir-1 levels were measured in infected cells, or the axis should be labeled something like ‘post-treatment’ or similar.*

We thank the reviewer for identifying this mistake. We have corrected **Figures 1H-I** to now read “24 h post-treatment” to reflect the experimental design and consistency of other figures.

7. *The pathway overview figure (Figure 4D) was very helpful – I am glad it was included, and I understand why it was the last figure of the paper, but I actually looked for something like this right away when starting to read the results, mostly in order to interpret the Western Blot results and how they matched the expectations. But I think it helped me then follow the other results (esp. Figure 1) better too. Maybe it could be introduced early on somehow? Or an adjusted version of it maybe? Just a thought because it would help understand the role of the inhibitors quickly (more so than the text). Figure 4D could be a panel in figure 1 of the paper and then the final figure could somehow link it back to the bigger organismal level - the strategy of mosquito feeding on a treated sugar bait, upregulation of RNAi and Jak/STAT, and reduced ZIKV transmission (?), assuming these mechanisms can be clarified a bit more.*

We are glad that the reviewer found our schematic helpful but recognize that the addition of other schematics may be helpful in presenting our proposed signaling mechanism and the function of our proposed drugs. We have added a schematic of the IIS pathway and drug function as **Figure 1A**. We have also made Figure 4D into its own separate figure as **Figure 6** which is highlighted in the discussion.

We have added the following statements for clarity:

- “(A) Insulin/ insulin-like growth factor I signaling (IIS) pathway schematic and proposed effect of DMAQ-B1 and AKT inhibitor VIII on downstream transcription factor activity.” (**Lines 752-754**)
- “Figure 6: Selective targeting of insulin signaling in *Ae. aegypti* impacts canonical antiviral responses that can effectively reduce ZIKV replication and likelihood of transmission. Schematic of proposed mechanism of antiviral action mediated by DMAQ-B1 and AKT inhibitor VIII during ZIKV infection through simultaneous induction of antiviral RNAi and JAK/STAT signaling.” (**Lines 835-839**)

8. *Line 100: reference 40 is also relevant here as one of the first to implicate JAK/STAT in ZIKV antiviral responses – none of the cited references 17-19 seem to be from mosquito-based studies (?).*

We thank the reviewer for identifying the discrepancy. We now reference Angleró-Rodríguez et al., within this section of the text.

9. *“Also, Figure 3G-I should be moved up since it these panels are mentioned first in the text.”*

We thank the reviewer for identifying this inconsistency and have revised the manuscript to discuss this figure, which is now Figure 4, in the order the panels are presented.

We have added the following statements:

- “At 7 d p.i. infection prevalence was significantly reduced in combined-treated midgut and salivary gland tissues but not carcasses (**Figs. 4A-C**). The titer of ZIKV-positive mosquitoes at 7 d p.i., however, was only reduced in the midguts of combined treated mosquitoes with an approximate 30-fold reduction. There was no difference in titer in carcass and salivary glands (**Figs. 4D-F**). By 11 d p.i., both infection prevalence (**Figs. 4G-I**), and viral titer (**Figs. 4J-L**) were significantly reduced across tissues in mosquitoes treated with individual and combined drug treatment relative to vehicle control. Titers in ZIKV-positive mosquitoes were reduced by ~42-fold in carcass, ~15-fold in midgut, and ~24-fold in salivary glands (**Figs. 4J-L**).” (**Lines 247-261**)
